# Optimizing detection of Alzheimer’s disease in mild cognitive impairment: a 4-year biomarker study of mild behavioral impairment in ADNI and MEMENTO

**DOI:** 10.1186/s13024-023-00631-6

**Published:** 2023-07-29

**Authors:** Zahinoor Ismail, Rebeca Leon, Byron Creese, Clive Ballard, Philippe Robert, Eric E. Smith

**Affiliations:** 1 Department of Psychiatry, Cumming School of Medicine, University of Calgary, 3330 Hospital Dr NW, Calgary, AB T2N 4N1 Canada; 2 Cumming School of Medicine, Hotchkiss Brain Institute, University of Calgary, 3330 Hospital Dr NW, Calgary, AB T2N 4N1 Canada; 3 Department of Clinical Neurosciences, Cumming School of Medicine, University of Calgary, 3330 Hospital Dr NW, Calgary, AB T2N 4N1 Canada; 4 Department of Community Health Sciences, Cumming School of Medicine, University of Calgary, 3330 Hospital Dr NW, Calgary, AB T2N 4N1 Canada; 5 O’Brien Institute for Public Health, Cumming School of Medicine, University of Calgary, 3280 Hospital Dr NW, Calgary, AB T2N 4Z6 Canada; 6 Department of Clinical and Biomedical Sciences, Faculty of Health and Life Sciences, University of Exeter, B3183, Exeter, EX1 2HZ UK; 7 CoBTeK, Université Côte d’Azur, Côte d’Azur, France

**Keywords:** Alzheimer’s disease, Mild cognitive impairment, Prodromal disease, Neuropsychiatric symptoms, Mild behavioral impairment, Biomarkers, Amyloid, Tau, Neurodegeneration, ATN, Cerebrospinal fluid

## Abstract

**Background:**

Disease-modifying drug use necessitates better Alzheimer disease (AD) detection. Mild cognitive impairment (MCI) leverages cognitive decline to identify the risk group; similarly, mild behavioral impairment (MBI) leverages behavioral change. Adding MBI to MCI improves dementia prognostication over conventional approaches of incorporating neuropsychiatric symptoms (NPS). Here, to determine if adding MBI would better identify AD, we interrogated associations between MBI in MCI, and cerebrospinal fluid biomarkers [β-amyloid (Aβ), phosphorylated-tau (p-tau), and total-tau (tau)-ATN], cross-sectionally and longitudinally.

**Methods:**

Data were from two independent referral-based cohorts, ADNI (mean[SD] follow-up 3.14[1.07] years) and MEMENTO (4.25[1.40] years), collected 2003–2021. Exposure was based on three-group stratification: 1) NPS meeting MBI criteria; 2) conventionally measured NPS (NPSnotMBI); and 3) noNPS. Cohorts were analyzed separately for: 1) cross-sectional associations between NPS status and ATN biomarkers (linear regressions); 2) 4-year longitudinal repeated-measures associations of MBI and NPSnotMBI with ATN biomarkers (hierarchical linear mixed-effects models-LMEs); and 3) rates of incident dementia (Cox proportional hazards regressions).

**Results:**

Of 510 MCI participants, 352 were from ADNI (43.5% females; mean [SD] age, 71.68 [7.40] years), and 158 from MEMENTO (46.2% females; 68.98 [8.18] years). In ADNI, MBI was associated with lower Aβ42 (standardized β [95%CI], -5.52% [-10.48-(-0.29)%]; *p* = 0.039), and Aβ42/40 (*p* = 0.01); higher p-tau (9.67% [3.96–15.70%]; *p* = 0.001), t-tau (7.71% [2.70–12.97%]; *p* = 0.002), p-tau/Aβ42 (*p* < 0.001), and t-tau/Aβ42 (*p* = 0.001). NPSnotMBI was associated only with lower Aβ42/40 (*p* = 0.045). LMEs revealed a similar 4-year AD-specific biomarker profile for MBI, with NPSnotMBI associated only with higher t-tau. MBI had a greater rate of incident dementia (HR [95%CI], 3.50 [1.99–6.17; *p* < 0.001). NPSnotMBI did not differ from noNPS (HR 0.96 [0.49–1.89]; *p* = 0.916). In MEMENTO, MBI demonstrated a similar magnitude and direction of effect for all biomarkers, but with a greater reduction in Aβ40. HR for incident dementia was 3.93 (*p* = 0.004) in MBI, and 1.83 (*p* = 0.266) in NPSnotMBI. Of MBI progressors to dementia, 81% developed AD dementia.

**Conclusions:**

These findings support a biological basis for NPS that meet MBI criteria, the continued inclusion of MBI in NIA-AA ATN clinical staging, and the utility of MBI criteria to improve identification of patients for enrollment in disease-modifying drug trials or for clinical care.

## Background

Inefficiencies and failures in the Alzheimer disease (AD) clinical trial program for disease-modifying therapies (DMTs) have been attributed to suboptimal detection of early phase illness [1]. Imprecise case ascertainment of prodromal AD based on standard clinical assessment requires further investigation. However, detailed neuropsychological testing and imaging, and cerebrospinal fluid (CSF) or positron emission tomography (PET) studies are laborious, expensive, and not universally available [2–7]. High screen-failure rates inflate costs, rendering some trials infeasible. Simple, inexpensive, and scalable proxy markers that improve AD detection in participants with mild cognitive impairment (MCI) are needed to reduce biomarker screen failures and improve trial efficiency. Further, in clinical care, as AD monoclonal antibody DMTs become available for use, finding clinical proxy markers that would help better identify ATN-framework-consistent AD is also an unmet need.

Neuropsychiatric or mental health symptoms (NPS) in older adults can initially emerge early in the disease course; 30% of AD cases present with NPS in advance of a cognitive diagnosis [8]. Mild Behavioral Impairment (MBI) is a syndrome that exploits this early manifestation of NPS to identify a high-risk group for incident cognitive decline and dementia [9, 10]. The ISTAART-AA criteria for MBI stipulate that NPS must emerge de novo in later life and persist for at least 6 months to qualify. This behavioral risk group is also described in the NIA-AA research framework for AD [10, 11]. In NIA-AA Stage 2, i.e., normal cognition or subjective cognitive decline (SCD), the staging description indicates that while cognition is the core feature, mild neurobehavioral symptoms, which have a recent onset and which persist, may coexist and may even be the primary complaint in some. In Stage 3, i.e., MCI, the framework indicates that while cognitive impairment is the core clinical criterion, neurobehavioral disturbance may be prominent in the clinical presentation [10, 11]. This linkage between the NIA-AA biological framework for AD and MBI provides further impetus to explore ATN biomarker correlates of neurobehavioral symptoms.

It is important to emphasize that MBI must be interpreted in the context of cognitive risk. MBI is not a competing construct or alternative to MCI, interpreted absent cognitive status, but is a complementary or add-on marker of risk, modifying the risk estimate for a specific cognitive status (i.e., NC, SCD, or MCI) [11–13]. Using these criteria, studies have demonstrated a significantly higher incidence rate of cognitive decline and dementia across cognitive categories in participants with MBI compared to participants with no NPS or with NPS not meeting MBI criteria (NPSnotMBI) [12–25]. Specific to MCI, recent findings showed that when participants were stratified by NPS status (i.e., noNPS, NPSnotMBI, MBI), MBI had a higher progression rate to dementia, and a lower reversion rate to normal cognition (CN) [13]. These findings demonstrate the utility of behavioral-risk stratification (represented by MBI) in conjunction with cognitive-risk stratification (represented by MCI) to improve specificity, and highlight the advantage of the MBI framework over conventional NPS models. However, biomarker confirmation of AD status in MCI progressors is required to better understand these associations, and to move this approach forward into clinical trial recruitment and clinical care.

Preliminarily, plasma, CSF, and PET data have demonstrated mostly cross-sectional associations between MBI and amyloid and p-tau [26–28]. Definitive studies are still required. Here, our aims were to determine if combining a behavioral assessment (informed by MBI criteria) together with a cognitive assessment (informed by MCI criteria) would identify a subgroup with: 1) greater baseline AD pathology (β-amyloid42, p-tau, t-tau); 2) greater change in AD biomarker levels over 4 years; and 3) greater 4-year incidence rate of dementia, specifically AD. The goal was not to determine if adding MBI to biomarkers would improve the performance of the biomarker models, but rather to determine if pre-screening with MBI could improve AD detection and prognostication via subsequent biomarker analysis, and improve our mechanistic understanding of the behavioral prodrome of AD.

## Methods

### ADNI and MEMENTO

Primary testing was in the Alzheimer’s Disease Neuroimaging Initiative (ADNI), a partnership involving multiple centers across North America with the goal of tracking participants through periods of cognitive decline and dementia. Launched in 2003, ADNI continues to evaluate biomarker, neuroimaging, and neuropsychological status in participants. All data used in this study were obtained prior to December 2021. Only participants with MCI were included in our study, to represent Stage 3 of the NIA-AA framework.

Validation was in the French MEMENTO cohort study [29], which recruited 2323 memory clinic patients with subjective cognitive complaints between April 2011 and June 2014. Participants were ≥ 60 years old, and MCI was defined as performance 1.5 SD below age and education adjusted norms in ≥ 1 cognitive domain (memory, language, praxis, visuospatial or executive function). Participants underwent clinical and neuropsychological assessments, brain MRI, and were reassessed at 6-month intervals. A subset of MEMENTO participants had CSF biomarker sampling in addition to regular assessments. Similarly, only participants with MCI were included.

### Sample

**Fig. 1:**
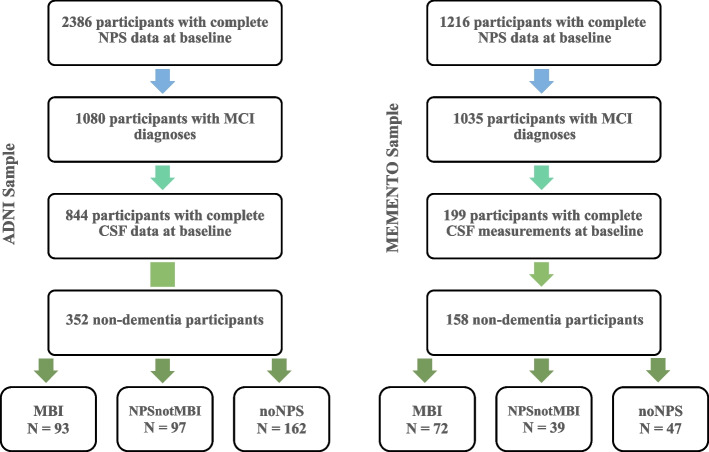
Flowchart showing how the sample populations were obtained from the ADNI and MEMENTO datasets

The study flowchart is shown in Fig. 1. Participants were included if they had complete Neuropsychiatric Inventory (NPI) or Neuropsychiatric Inventory Questionnaire (NPI-Q) [30] data for the first (baseline), and second (1-year) visits. A total of 510 MCI participants were included in the analysis, all with CSF measures at baseline. Biomarkers included β-amyloid42 (Aβ42), β-amyloid40 (Aβ40), p-tau, t-tau, and Aβ42/Aβ40, p-tau/Aβ42, and t-tau/Aβ42 ratios.

### MBI case ascertainment

NPI or NPI-Q scores were used to determine NPS status. The NPI and NPI-Q consist of 12 NPS domains, scored for frequency and/or severity over a 1-month reference range. NPI/NPI-Q domains were used to derive MBI domains using a published algorithm [31]: MBI motivation/drive (apathy) from apathy/indifference; MBI emotional regulation (mood/anxiety symptoms) from the sum of depression, anxiety, and elation/euphoria; MBI impulse control (agitation, aggression, impulsivity) from the sum of irritability, agitation/aggression, and aberrant motor behavior; MBI social inappropriateness (impaired social cognition) from disinhibition; and MBI abnormal thoughts/perception (psychotic symptoms) from delusions/hallucinations. Converted NPI/NPI-Q scores for these 10 domains were then transformed into the 5 MBI domains; sleep and appetite changes were not included in the algorithm. Baseline and one-year visits were used to operationalize the MBI symptom persistence criterion. For each visit, a transformed NPS total score > 0 was classified as NPS + . Two consecutive NPS + visits were classified as MBI (i.e., persistent NPS); and a conventional NPS + score at one visit was classified as NPSnotMBI (i.e., transient NPS). Two consecutive NPS- visits were classified as noNPS.

### Statistical analysis

Basic demographic information was abstracted (age, sex, education, and Mini-Mental State Examination (MMSE) score). NPS groups (MBI, NPSnotMBI) were compared against the noNPS group using Chi-squared tests (categorical variables) and independent *t*-tests (continuous variables).

Linear regressions were fitted to determine cross-sectional associations between NPS status as independent variable, and CSF biomarkers as continuous dependent variables. Logarithmic transformations were applied to CSF biomarker measures due to skewness. All models adjusted for age, sex, education, MMSE scores and the source of NPS (NPI or NPI-Q). MBI status was coded using dummy variables, corresponding to MBI, NPSnotMBI, and noNPS groups. Contrasts were set up for comparisons against the noNPS group. NPS source was coded using dummy variables, corresponding to NPI for both visits, NPI-Q for both visits, or a combination of NPI and NPI-Q; NPI for both visits was the reference.

Hierarchical linear mixed-effects (LME) models were implemented to assess the longitudinal relationship between NPS profile and CSF biomarkers over the span of 4 years. Annual NPS measure was predictor, and concurrent annual measure of CSF biomarker level was outcome. Time-varying covariates were leveraged to capture stable between-person NPS differences (consistent with MBI symptom persistence) and within-person visit-to-visit variability (consistent with impersistent or transient NPS, i.e., NPSnotMBI). Using a person-centered approach, the between-person NPS predictor was calculated as the mean total NPS score across all visits, and the within-person NPS predictor was calculated as the NPS score for each visit minus the between-person NPS score [32]. Participant ID was modeled as a random effect. Fixed effects include age, sex, education, NPS (between- and within-person), MMSE, time, diagnosis, and NPS scale (NPI, NPI-Q, combination).

Kaplan–Meier survival curves were generated to compare dementia-free survival probability for the 3 groups (MBI, NPSnotMBI, noNPS). Cox proportional hazards regressions were utilized to compare rates of progression from MCI to dementia as a function of NPS status (MBI, NPSnotMBI, noNPS), while controlling for age, sex, years of education, and MMSE score at baseline. ADNI did not provide information on dementia subtypes, but MEMENTO did. Thus, dementia progressors in MEMENTO were further reported by percentage of participants in each dementia subtype. For the ADNI dataset, statistical analyses were performed using R (version 4.2.2). For the MEMENTO validation dataset, analyses were performed using R (version 4.1.3) on the Dementias Platform UK Data Portal (DPUK). Assumptions of linear regression were satisfied, assessed using the *ggfortify* package in R. Linearity was assessed with a Martingale residuals plot. Assumptions for Cox proportional hazards were satisfied, tested using a Schoenfeld test and linearity was assessed with a Martingale residuals plot from the *survival* and *survminer* packages in R.

## Results

### ADNI

The ADNI sample comprised 352 participants (43.5% females; mean [SD] age, 71.68 [7.40] years). NPS group was determined using the NPI in 235 participants, and NPI-Q in 117 participants. Demographic information is shown in Table 1. Linear regressions showed that compared to noNPS, MBI was associated with lower CSF Aβ42 level and Aβ42/40 ratio, higher p-tau and t-tau levels, and higher t-tau/Aβ42 and p-tau/Aβ42 ratios; NPSnotMBI was associated only with lower Aβ42/40 (Table 2).

**Table 1:**
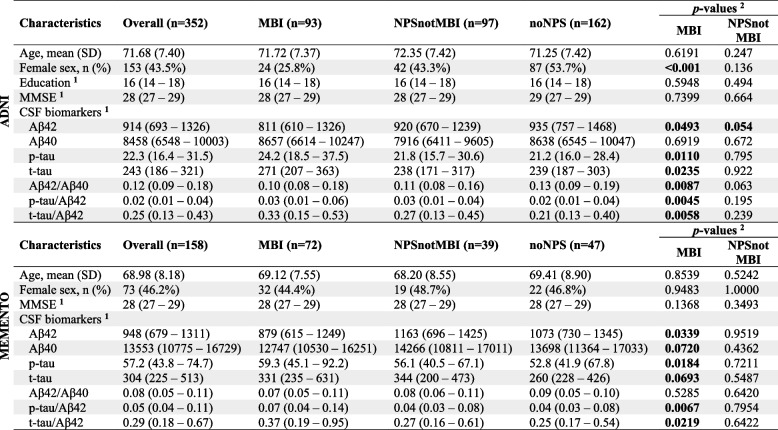
Sample characteristics at baseline

^1^Median values and interquartile ranges are shown. Cerebrospinal fluid biomarkers values are shown raw. ^2^t-tests (two-tailed) were performed on log-transformed values. MBI and NPSnotMBI compared to noNPS. Abbreviations: MBI=Mild Behavioural Impairment; MMSE=Mini-Mental State Exam

**Table 2:**
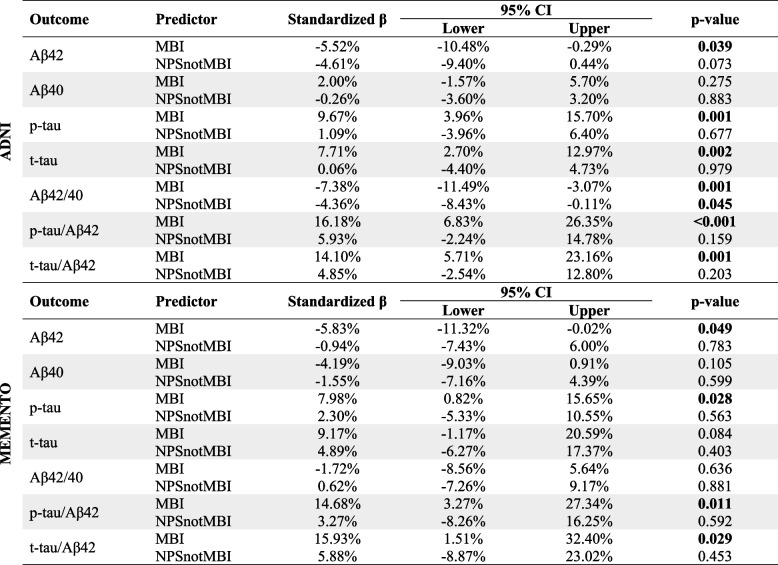
Association between NPS group and CSF biomarkers modeled using linear regression models

Beta coefficients represent the estimate percent difference in the CSF marker compared to the noNPS groups. Models adjusted for age, sex, education, and source of NPS

Longitudinal biomarker measures were available in 161/352 participants at two years, 99/352 at three years, and 216/352 at four years. LME models tracking the sample over 4 years showed that higher between-person NPS differences (i.e., MBI) were associated with lower CSFAβ42 level and Aβ42/40 ratio, higher CSF p-tau and t-tau levels, and higher p-tau/Aβ42 and t-tau/Aβ42 ratios. Within-person NPS variability (i.e., NPSnotMBI) only associated with slightly higher t-tau (Table 3).

**Table 3:**
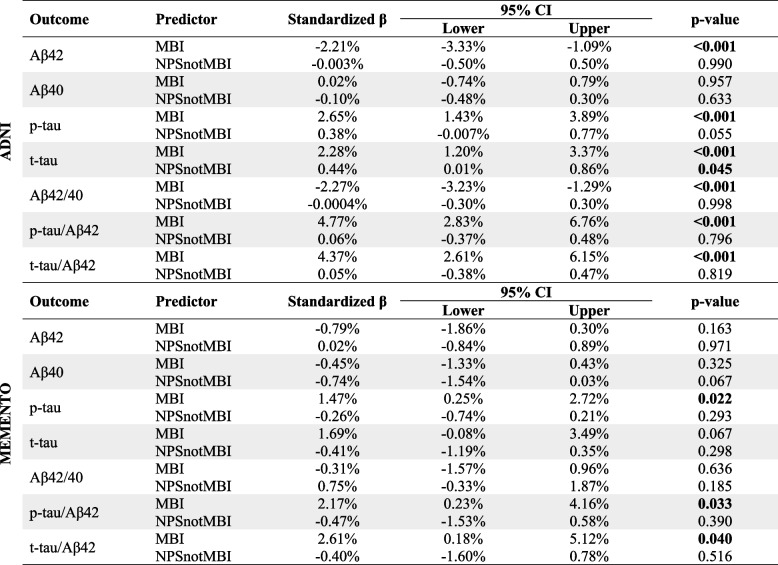
Association between NPS group and CSF biomarkers in a span of 4 years using linear mixed effect models

Beta coefficients represent the estimate percent difference in the CSF marker compared to the noNPS groups. Models adjusted for age, sex, education, and source of NPS

**Fig. 2:**
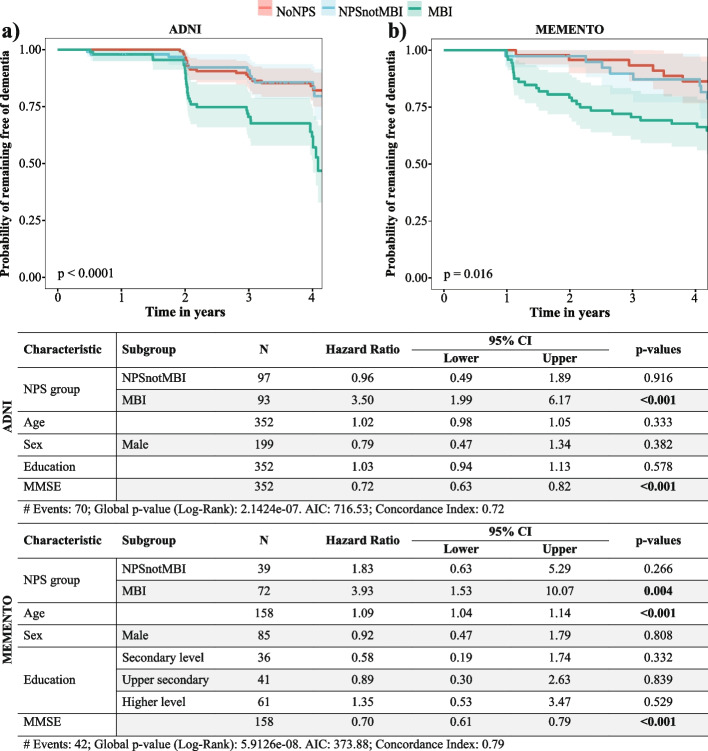
Kaplan-Meir curve and Cox proportional hazards ratios for incident dementia

During follow-up (mean [SD], 3.14 [1.07] years), 70 participants progressed from MCI to dementia. Kaplan–Meier survival curves demonstrated significantly lower dementia-free survival at 4 years in the MBI group relative to NPSnotMBI and noNPS (Fig. 2a). Cox regression showed that individuals with MBI had a 3.5-fold greater incidence rate of dementia while NPSnotMBI did not differ from noNPS (HR = 0.96).

### MEMENTO

The MEMENTO sample comprised 158 participants (46.2% females; mean [SD] age, 68.98 [8.18] years). NPS group was determined using NPI alone. Demographic information is shown in Table 1. Linear regressions showed that compared to noNPS, MBI was associated with lower CSF Aβ42 level, higher p-tau, and higher p-tau/Aβ42 and t-tau/Aβ42 ratios; NPSnotMBI did not significantly differ from noNPS on any of the measures (Table 2).

Longitudinal CSF measures were available in 77/158 participants at two years, 47/158 at three years, and 72/158 at four years. LME models showed that MBI was associated with higher CSF p-tau levels, and higher p-tau/Aβ42 and t-tau/Aβ42 ratios; NPSnotMBI was not associated with any of the measures (Table 3).

During follow up (mean [SD], 4.25 [1.40] years), 42 participants progressed from MCI to dementia. Kaplan–Meier survival curves demonstrated significantly lower dementia-free survival at 4 years in the MBI group relative to the NPSnotMBI and noNPS groups (Fig. 2b). The Cox regression showed that individuals with MBI had a 3.93-fold greater rate of incident dementia from MCI; NPSnotMBI had a 1.83-fold greater rate, not significantly different from noNPS. Of the dementia progressors in the MEMENTO sample, 34/42 (81.0%) developed AD dementia, 3/42 (7.1%) developed Lewy body dementia, 2/42 (4.8%) developed mixed dementia, and 3/42 (7.1%) had unknown dementia subtype.

## Discussion

In this 4-year study of 510 participants with MCI, both the ADNI test sample and the MEMENTO validation sample demonstrated that MBI was cross-sectionally associated with AD biomarkers, and longitudinally associated with changes in these biomarkers, consistent with AD. Further, survival analyses found MBI to have a higher incidence rate of dementia than comparator groups and in the MEMENTO sample, 81% of dementia progressors developed AD. These findings demonstrate that applying the MBI criteria to MCI improves identification of individuals with positive AD biomarkers who are at higher risk for progression to AD dementia.

### Cross sectional analysis

Across studies, MBI was associated with biomarker profiles consistent with prodromal AD. The one exception was MEMENTO in which there was no association with lower Aβ42/40 ratio, driven by lower Aβ40 levels (standardized β, -4.19% in MBI; -1.55% in NPSnotMBI). These Aβ40 differences were not seen in ADNI (standardized β, + 2.00% in MBI; -0.26% in NPSnotMBI). Possible explanations for lower Aβ40 include the presence of neuroinflammation [33] or amyloid angiopathy [34], and nicotine exposure [35]. Participant selection differences between studies may account for the divergent Aβ40 results. ADNI inclusion criteria are more restrictive, e.g., with respect to concurrent vascular burden[36]. MEMENTO is a study of real-world memory clinic patients, more heterogeneous than ADNI; participants likely had a greater vascular disease burden [37], and possibly higher nicotine use. Thus, MEMENTO may harbor more potential contributors to lower Aβ40.

On the other hand, previous research has found a significant relationship between lower Aβ40 and a more rapid annual decline in MMSE in AD progressors [38, 39]. It has been hypothesized that those with low levels of Aβ40 may have more advanced amyloid plaque pathology, sequestering both Aβ42 and Aβ40 [39]. The Kaplan–Meier survival curves show a faster separation of MBI from the other two groups in MEMENTO vs ADNI, in line with the Aβ40 hypothesis. More research into this finding is required, however, the similarity between ADNI and MEMENTO for the remainder of the findings is reassuring.

In contrast, NPSnotMBI was only associated with lower Aβ42/40 ratio in ADNI. Although, the standardized β-coefficient for the ADNI Aβ42 level was comparable to MBI (-4.61% vs -5.52%), despite not meeting the threshold for significance. P-tau levels were substantially different (standardized β, + 9.67% in MBI, + 1.09% in NPSnotMBI). The NPSnotMBI group may include persons with behavioral and cognitive symptoms secondary to life stressors or other causes, who are less likely to harbor AD. This finding accords with a previous systematic review of CSF studies in NPS, suggesting that noise in NPS measurement may result in inconsistent results [40].

The few previous cross-sectional studies on biomarker associations with MBI have been more consistent, although none were completed in an exclusively MCI sample. The Canadian TRIAD study and the Swedish BioFINDER2 study converged regarding MBI associations with biomarkers in CN participants. TRIAD demonstrated a correlation between MBI severity and greater global and striatal amyloid-PET tracer binding; Biofinder2 demonstrated associations between MBI and entorhinal and hippocampal tau-PET tracer uptake, and elevated CSF tau in amyloid positive participants [26, 27]. This finding was very recently replicated with plasma p-tau181 [41]. Another study, in a mixed sample of NC and MCI participants, reported an association between lower plasma Aβ42/Aβ40 ratio and higher MBI score [28]. These findings are congruent with the a priori development goal of MBI to increase signal and reduce noise when using NPS to identify preclinical and prodromal AD [12, 42].

The MBI-related differences in p-tau/Aβ42 and t-tau/Aβ42 in ADNI and MEMENTO are meaningful. These ratios are important biomarkers for detecting prodromal AD in MCI, and for AD prognostication. Both p-tau/Aβ42 and t-tau/Aβ42 ratios have demonstrated high concordance with amyloid PET classification, predicting greater 2-year clinical decline in patients with MCI [43]. A recent study that included all three CSF markers determined p-tau/Aβ42 as the most accurate predictor of imminent progression to AD (sensitivity 82.9%, specificity 90%) over a mean time of 26.07 months [44]. Thus, the biomarker profiles from both ADNI and MEMENTO align with AD-related changes for amyloid, tau, and neurodegeneration in association with MBI but not with the comparator groups. These findings support a biological basis for the NPS that meet MBI criteria, the inclusion of MBI in the ATN framework, and the utility of the MBI criteria for identifying prodromal AD.

### Longitudinal analyses – LME models

That MBI in MCI was associated with AD-related changes over 4 years, while NPSnotMBI was not, is an important and novel finding. Longitudinal data for MBI and AD biomarkers in dementia-free individuals are scarce, and absent in an MCI-only sample. A mixed CN/MCI ADNI study reported an association between MBI and a 2-year increase in plasma neurofilament light, a marker of axonal loss or neurodegeneration [45]. Very recently, a related study utilizing ADNI plasma p-tau181 samples found a similar increase in p-tau over time in association with MBI, as well as greater decline in memory and executive function, vs noNPS and NPSnotMBI comparators [41]. Our CSF study across two independent cohorts is the most definitive evidence thus far, demonstrating that refining NPS measurement can enrich samples for AD. In our study, longitudinal results were not identical in ADNI and MEMENTO. In fact, we would not expect them to be. ADNI is a restricted cohort that mimics a clinical trial and MEMENTO comprises real world memory clinic patients – we would expect more heterogeneity in MEMENTO. Further, due to the smaller sample size in MEMENTO, the MBI estimate was not precise enough to statistically differ from NPS-not-MBI, despite the difference in magnitude and direction.

### Survival analyses – Kaplan–Meier and Cox regressions

Across ADNI and MEMENTO, dementia-free survival was significantly lower in the MBI group versus the NPSnotMBI and noNPS groups. Cox regressions demonstrated that MBI had a significantly and substantially higher rate of incident dementia compared to noNPS, while NPSnotMBI did not differ from noNPS. These survival analysis results conform with the findings from the cross-sectional and longitudinal biomarker analyses. Findings also align with the bulk of the MBI literature comprising epidemiological or cohort studies with MBI as exposure, and outcomes of cognition, function, or risk marker. Consistently, whether in CN, MCI, or mixed dementia-free samples, or whether in clinical, community, or even online cohort studies, the data have consistently shown that selecting NPS in accordance with MBI criteria identifies a group with greater baseline risk [37, 46–52], and/or greater risk for incident MCI and dementia [12–20, 41, 53, 54] than the comparator groups.

The current study extends previous work to demonstrate clear associations between MBI and the full palette of CSF biomarkers in the NIA-AA research framework for AD [11]. However, the translation to clinical care or clinical trial recruitment is imperative. In these observational cohorts, we operationalized the MBI cardinal criteria of de novo symptom emergence in later life and symptom persistence for ≥ 6 months by 1) including only participants with no formal psychiatric and neurodevelopmental conditions; and 2) ensuring NPS were present at two consecutive visits. This approach is feasible in research cohorts, where multiple visits are available for selection and stratification, but not at an initial clinic visit or clinical trial screening assessment.

Single-visit alternatives to implement MBI criteria are required. In anticipation of this issue, ISTAART developed the mild behavioral impairment checklist (MBI-C) as the case ascertainment instrument to measure MBI in accordance with the criteria. The MBI-C assesses NPS across the domains of MBI, with explicit stipulations that symptoms represent change and persist, language echoed in the ATN framework [11]. Multiple validations of the MBI-C have been published [55–63], and it has been used in several published epidemiological and biomarker studies [14, 18, 19, 26, 27, 49, 51, 62, 64]. However, more research is required, and the methods used in the current study need replication in different and more diverse cohorts using the MBI-C as the NPS measure. However, our findings are novel and meaningful, for the first time showing clear associations between MBI in MCI and cross-sectional and longitudinal CSF-measured ATN biomarkers, supporting the utility of MBI to improve specificity of MCI in detection of prodromal AD [13]. The clinical translation of these findings would be to include an assessment of MBI (e.g., MBI-C at a single clinic visit or NPI over two clinic visits) in conjunction with a standard measure of cognition (e.g., MMSE). The presence of MBI would influence the interpretation of the cognitive measure and change the estimate for presence of AD biomarkers at the time of the visit, and the potential change of the biomarkers and cognition over time.

### Limitations

MBI symptoms were based on NPI-measured NPS at study visits. Transient symptoms present only within the one-month reference range preceding the visit could be included in MBI case status, resulting in false positives. Conversely, the breadth of MBI symptoms and behaviors may not have been captured with the current approach, resulting in false negatives. Both types of errors could reduce the magnitude of effect. The exclusion of participants with a history of psychiatric conditions could also be a limitation, especially if symptoms were of relatively recent onset but still diagnosed as formal psychiatric conditions. It is conceivable that some of these excluded participants had MBI rather than a psychiatric disorder. Our MBI case status reflected global NPS burden, rather than individual MBI domains (i.e., apathy, affect, impulsivity, social inappropriateness, and psychosis). Persistent NPS using our operational definition could consist of symptoms from different domains. Subsequent domain-specific analyses are required, which will require substantially larger samples. Finally, while ADNI and MEMENTO are similar enough to compare in this analysis, different inclusion criteria, participant characteristics, and comorbidities could confound the relationship between NPS status and biomarkers. Nonetheless, the similarities are reassuring.

## Conclusions

Mental health symptoms are important links to cognitive health and better assessment and classification of these symptoms can result in more accurate dementia detection. Our findings suggest AD proteinopathies may be part of the neurobiology of later-life onset and persistent NPS that meet MBI criteria. The finding that 81% of MBI progressors to dementia developed AD support the continued inclusion of MBI in the ATN framework and clinical staging. Results are congruent with the a priori goals in development of the MBI criteria and have implications for research methodology, clinical trial recruitment, drug development, and clinical care.

## Data Availability

Data from ADNI are available by request, and authors will share data cleaning script with interested parties. MEMENTO data require a specific application to the MEMENTO secretariat and analyses are completed on the DPUK platform. Authors will also share R script for MEMENTO data upon request.

## Appendix

**Table 4:** A list of Memento collaborators

Name	Degree	Location	Role
Michèle Allard	MD, PhD	Memory Resource and Research Centre of Bordeaux, CHU de Bordeaux, Hôpital Xavier Arnozan, F-33000, Bordeaux, France	Co-investigator
Sandrine Andrieu	MD, PhD	Memory Resource and Research Centre of Toulouse, CHU de Toulouse, Hôpital La Grave-Casselardit, F-31000, Toulouse, France	Co-investigator
Pierre Anthony	MD, PhD	Memory Resource and Research Centre of Colmar, Hôpitaux Civils de Colmar, F-68000, Colmar, France	Co-investigator
Christine Astier	MD	Memory Resource and Research Centre of Strasbourg, Hôpitaux Universitaires de Strasbourg, F-67000, Strasbourg, France	Co-investigator
Alexandre Augier	MD, PhD	Memory Clinic, Hôpital Avicenne, AP-HP, Hôpitaux Universitaires Paris-Seine-Saint-Denis, F-93009, Bobigny, France	Co-investigator
Nicolas Auguste	MD	Memory Resource and Research Centre of Saint-Etienne, CHU de Saint-Etienne, Hôpital de la Charité, F-42000, Saint-Etienne, France	Co-investigator
Sophie Auriacombe	MD, PhD	Memory Resource and Research Centre of Bordeaux, CHU de Bordeaux, Hôpital Pellegrin, F-33000, Bordeaux, France	Co-investigator
John Avet	MD, PhD	Memory Resource and Research Centre of Saint-Etienne, CHU de Saint-Etienne, Hôpital Nord, F-42000, Saint-Etienne, France	Co-investigator
Olivier Bailon	MD, PhD	Memory Clinic, Hôpital Avicenne, AP-HP, Hôpitaux Universitaires Paris-Seine-Saint-Denis, F-93009, Bobigny, France	Co-investigator
Fabrice-Guy Barral	MD	Memory Resource and Research Centre of Saint-Etienne, CHU de Saint-Etienne, Hôpital Nord, F-42000, Saint-Etienne, France	Co-investigator
Jean Barré	MD	Memory Resource and Research Centre of Angers, CHU d’Angers, F-49000, Angers	Co-investigator
Annick Barthelaix	MD, PhD	Memory Resource and Research Centre of Angers, CHU d’Angers, F-49000, Angers	Co-investigator
Catherine Bayle	MD	Memory Resource and Research Centre of Paris Broca, AP-HP, Paris, France	Co-investigator
Olivier Beauchet		Memory Resource and Research Centre of Angers, CHU d’Angers, F-49000, Angers	Co-investigator
Catherine Belin	MD, PhD	Memory Clinic, Hôpital Avicenne, AP-HP, Hôpitaux Universitaires Paris-Seine-Saint-Denis, F-93009, Bobigny, France	Co-investigator
Samia Belkacem	MD	Institute of Memory and Alzheimer's Disease (IM2A), Centre for NeuroImaging Research (CENIR), Brain and Spine Institute (ICM), UMR S 1127, Department of Neurology, AP-HP, Pitié-Salpêtrière University Hospital, Sorbonne Universities, Pierre et Marie Curie University, Paris, France	Co-investigator
Douraied Ben Salem	MD, PhD	Memory Resource and Research Centre of Brest, CHRU de Brest, F-29000, Brest, France	Co-investigator
Karim Bennys	MD	Memory Resource and Research Centre of Montpellier, CHU de Montpellier, Hôpital Gui de Chauliac, F-34000, Montpellier, France	Co-investigator
Géraldine Bera	MD	Laboratoire d'Imagerie Biomédicale, Sorbonne Universités, UPMC Univ Paris 06, Inserm U1146, CNRS UMR 7371, France NeuroSpin, I2BM, Commissariat à l'Energie Atomique, Paris, France	Co-investigator
Eric Berger	MD	Memory Resource and Research Centre of Besançon, CHU de Besançon, Hôpital Jean Minjoz, Hôpital Saint-Jacques, F-25000, Besançon, France	Co-investigator
Marc G Berger	MD, PhD	Memory Resource and Research Centre of Clermont-Ferrand, CHU de Clermont-Ferrand, F-63000, Clermont-Ferrand, France	Co-investigator
Emilie Bergouin	MD	Memory Resource and Research Centre of Dijon, CHU Dijon Bourgogne, Hôpital du Bocage, Hôpital de Champmaillot, F-21000, Dijon, France	Co-investigator
François Bertin-Hugault	MD	Memory Resource and Research Centre of Lyon, Hospices Civils de Lyon, Hôpital des Charpennes, F-69000, Lyon, France	Co-investigator
Guillaume Bertrand	MD	Memory Clinic, Hôpital Avicenne, AP-HP, Hôpitaux Universitaires Paris-Seine-Saint-Denis, F-93009, Bobigny, France	Co-investigator
François-Xavier Bertrand	MD, PhD	Memory Resource and Research Centre of Nantes, CHU de Nantes, F-44000, Nantes, France	Co-investigator
Catherine Beze	MD	Memory Resource and Research Centre of Center Region, CHRU de Tours, Hôpital Bretonneau, F-37000, Tours, France	Co-investigator
Valérie Boilet		Coordinating Centre, Inserm CIC-1401 Clinical Epidemiology, CHU de Bordeaux, F-33000, Bordeaux, France	Co-investigator
Stéphanie Bombois	MD, PhD	Memory Resource and Research Centre of Lille, CHRU de Lille, Hôpital Roger Salengro, F-59000, Lille, France	Co-investigator
Alain Bonafé	MD, PhD	Memory Resource and Research Centre of Montpellier, CHU de Montpellier, Montpellier, France	Co-investigator
Yasmina Boudali	MD	Memory Resource and Research Centre of Paris Broca, AP-HP, Paris, France	Co-investigator
Hatem Bouhladour	MD, PhD	Memory Resource and Research Centre of Besançon, CHU de Besançon, Hôpital Jean Minjoz, Hôpital Saint-Jacques, F-25000, Besançon, France	Co-investigator
Clémence Boully	MD	Memory Resource and Research Centre of Paris Broca, AP-HP, Paris, France	Co-investigator
Isabelle Bourdel-Marchasson	MD, PhD	Memory Resource and Research Centre of Bordeaux, CHU de Bordeaux, Hôpital Xavier Arnozan, F-33000, Bordeaux, France	Co-investigator
Vincent Bouteloup	PharmD	Coordinating Centre, Inserm CIC-1401 Clinical Epidemiology, CHU de Bordeaux, F-33000, Bordeaux, France	Co-investigator
Claire Boutet	MD	Institute of Memory and Alzheimer's Disease (IM2A), Centre for NeuroImaging Research (CENIR), Brain and Spine Institute (ICM), UMR S 1127, Department of Neurology, AP-HP, Pitié-Salpêtrière University Hospital, Sorbonne Universities, Pierre et Marie Curie University, Paris, France	Co-investigator
Serge Bracard	MD, PhD	Memory Resource and Research Centre of Nancy, CHU de Nancy, F-54000, Nancy, France	Co-investigator
Antoine Brangier	MD	Memory Resource and Research Centre of Angers, CHU d’Angers, F-49000, Angers	Co-investigator
Pierre-Yves Brillet	MD, PhD	Memory Clinic, Hôpital Avicenne, AP-HP, Hôpitaux Universitaires Paris-Seine-Saint-Denis, F-93009, Bobigny, France	Co-investigator
Laure Caillard	MD	Memory Resource and Research Centre of Paris Broca, AP-HP, Paris, France	Co-investigator
Fabienne Calvas	MD	Memory Resource and Research Centre of Toulouse, CHU de Toulouse, Hôpital Purpan, F-31000, Toulouse, France	Co-investigator
Agnès Camus	MD	Memory Resource and Research Centre of Dijon, CHU Dijon Bourgogne, Hôpital du Bocage, Hôpital de Champmaillot, F-21000, Dijon, France	Co-investigator
Vincent Camus	MD, PhD	Memory Resource and Research Centre of Center Region, CHRU de Tours, Hôpital Bretonneau, F-37000, Tours, France	Co-investigator
Sandrine Canaple	MD	Memory Resource and Research of Amiens, CHU Amiens Picardie, F-80000, Amiens, France	Co-investigator
Antoine Carpentier	MD, PhD	Memory Clinic, Hôpital Avicenne, AP-HP, Hôpitaux Universitaires Paris-Seine-Saint-Denis, F-93009, Bobigny, France	Co-investigator
Pascaline Cassagnaud	MD	Memory Resource and Research Centre of Lille, CHRU de Lille, Hôpital Roger Salengro, F-59000, Lille, France	Co-investigator
Françoise Cattin	MD	Memory Resource and Research Centre of Besançon, CHU de Besançon, Hôpital Jean Minjoz, Hôpital Saint-Jacques, F-25000, Besançon, France	Co-investigator
Ludivine Chamard	MD	Memory Resource and Research Centre of Besançon, CHU de Besançon, Hôpital Jean Minjoz, Hôpital Saint-Jacques, F-25000, Besançon, France	Co-investigator
Stéphane Chanalet	MD	Memory Resource and Research Centre of Nice, CHU de Nice, Hôpital Pasteur, F-06100, Nice, France	Co-investigator
Mathieu Chastan	MD	Memory Resource and Research Centre of Rouen, CLCC Henri Becquerel, Rouen, France	Co-investigator
Sophie Chauvelier	MD	Memory Resource and Research Centre of Paris Broca, AP-HP, Paris, France	Co-investigator
Valérie Chauvire	MD	Memory Resource and Research Centre of Angers, CHU d’Angers, F-49000, Angers	Co-investigator
Samia Cheriet	MD, PhD	Memory Resource and Research Centre of Toulouse, CHU de Toulouse, Hôpital Purpan, F-31000, Toulouse, France	Co-investigator
Anthony Clotagatide	MD	Memory Resource and Research Centre of Saint-Etienne, CHU de Saint-Etienne, Hôpital Nord, F-42000, Saint-Etienne, France	Co-investigator
Emmanuel Cognat	MD, PhD	Memory Resource and Research Centre of Paris Nord, AP-HP, Paris, France	Co-investigator
Lora Cohen	PhD	Memory Resource and Research Centre of Grenoble, CHU de Grenoble Alpes, Grenoble, France	Co-investigator
Jean-Marc Constans	MD, PhD	Memory Resource and Research of Amiens, CHU Amiens Picardie, F-80000, Amiens, France	Co-investigator
Marie-Hélène Coste	MD, PhD	Memory Resource and Research Centre of Lyon, Hospices Civils de Lyon, Hôpital des Charpennes, F-69000, Lyon, France	Co-investigator
Jean-Philippe Cottier	MD, PhD	Memory Resource and Research Centre of Center Region, CHRU de Tours, Hôpital Bretonneau, F-37000, Tours, France	Co-investigator
François Cotton	MD, PhD	Memory Resource and Research Centre of Lyon, Hospices Civils de Lyon, Hôpital des Charpennes, F-69000, Lyon, France	Co-investigator
Isabelle Couret	MD	Memory Resource and Research Centre of Montpellier, CHU de Montpellier, Hôpital Gui de Chauliac, F-34000, Montpellier, France	Co-investigator
Olivier-François Couturier	MD, PhD	Memory Resource and Research Centre of Angers, CHU d’Angers, F-49000, Angers	Co-investigator
Pascale Cowppli-Bony	MD, PhD	Memory Resource and Research Centre of Bordeaux, CHU de Bordeaux, Hôpital Pellegrin, F-33000, Bordeaux, France	Co-investigator
Véronique Cressot	MD	Memory Resource and Research Centre of Bordeaux, CHU de Bordeaux, Hôpital Xavier Arnozan, F-33000, Bordeaux, France	Co-investigator
Benjamin Crétin	MD	Memory Resource and Research Centre of Strasbourg, Hôpitaux Universitaires de Strasbourg, F-67000, Strasbourg, France	Co-investigator
Keren Danaila	MD	Memory Resource and Research Centre of Lyon, Hospices Civils de Lyon, Hôpital des Charpennes, F-69000, Lyon, France	Co-investigator
Jacques Darcourt	MD, PhD	Memory Resource and Research Centre of Nice, CLCC Antoine Lacassagne, Nice, France	Co-investigator
Jean-François Dartigues	MD, PhD	Memory Resource and Research Centre of Bordeaux, CHU de Bordeaux, Hôpital Pellegrin, F-33000, Bordeaux, France	Co-investigator
Ana-Maria Dascalita	MD, PhD	Memory Resource and Research Centre of Saint-Etienne, CHU de Saint-Etienne, Hôpital de la Charité, F-42000, Saint-Etienne, France	Co-investigator
Renaud David	MD, PhD	Memory Resource and Research Centre of Nice, CHU de Nice, Institut Claude Pompidou, F-06100, Nice, France	Co-investigator
Xavier De Petigny	MD	Memory Resource and Research Centre of Strasbourg, Hôpitaux Universitaires de Strasbourg, F-67000, Strasbourg, France	Co-investigator
Delphine De Verbizier-Lonjon	MD	Memory Resource and Research Centre of Montpellier, CHU de Montpellier, Hôpital Gui de Chauliac, F-34000, Montpellier, France	Co-investigator
Marielle Decousus	MD, PhD	Memory Resource and Research Centre of Saint-Etienne, CHU de Saint-Etienne, Hôpital Nord, F-42000, Saint-Etienne, France	Co-investigator
Isabelle Defouilloy	MD, PhD	Memory Resource and Research of Amiens, CHU Amiens Picardie, F-80000, Amiens, France	Co-investigator
Christine Delmaire	MD, PhD	Memory Resource and Research Centre of Lille, CHRU de Lille, Hôpital Roger Salengro, F-59000, Lille, France	Co-investigator
Julien Delrieu	MD	Memory Resource and Research Centre of Toulouse, CHU de Toulouse, Hôpital La Grave-Casselardit, F-31000, Toulouse, France	Co-investigator
Catherine Demuyinck	MD	Memory Resource and Research Centre of Strasbourg, Hôpitaux Universitaires de Strasbourg, F-67000, Strasbourg, France	Co-investigator
Vincent Deramecourt	MD, PhD	Memory Resource and Research Centre of Lille, CHRU de Lille, Hôpital Roger Salengro, F-59000, Lille, France	Co-investigator
Hervé Deramond	MD, PhD	Memory Resource and Research of Amiens, CHU Amiens Picardie, F-80000, Amiens, France	Co-investigator
Thomas Desmidt	MD, PhD	Memory Resource and Research Centre of Center Region, CHRU de Tours, Hôpital Bretonneau, F-37000, Tours, France	Co-investigator
Marie-Dominique Desruet	PharmD, PhD	Memory Resource and Research Centre of Grenoble, CHU de Grenoble Alpes, Grenoble, France	Co-investigator
Julien Detour		Memory Resource and Research Centre of Strasbourg, Hôpitaux Universitaires de Strasbourg, F-67000, Strasbourg, France	Co-investigator
Agnès Devendeville	MD	Memory Resource and Research of Amiens, CHU Amiens Picardie, F-80000, Amiens, France	Co-investigator
Mira Didic	MD, PhD	Memory Resource and Research Centre of Marseille, CHU de Marseille, Hôpital La Timone, F-13000, Marseille, France	Co-investigator
Maritchu Doireau	MD	Memory Resource and Research Centre of Bordeaux, CHU de Bordeaux, Hôpital Pellegrin, F-33000, Bordeaux, France	Co-investigator
Antonio Dos Santos	MD	Institute of Memory and Alzheimer's Disease (IM2A), Brain and Spine Institute (ICM), UMR S 1127, Department of Neurology, AP-HP, Pitié-Salpêtrière University Hospital, Sorbonne Universities, Pierre et Marie Curie University, Paris, France	Co-investigator
Patrice Douillet	MD	Memory Resource and Research Centre of Montpellier, CHU de Montpellier, Hôpital Gui de Chauliac, F-34000, Montpellier, France	Co-investigator
Foucaud Du Boisgueheneuc	MD	Memory Resource and Research Centre of Poitiers, CHU de Poitiers, Hôpital de La Milétrie, F-86000, Poitiers, France	Co-investigator
Delphine Dubail	MD	Memory Resource and Research Centre of Paris Broca, AP-HP, Paris, France	Co-investigator
Laure Ducroq-Ducastaing	MD	Memory Resource and Research Centre of Brest, CHRU de Brest, F-29000, Brest, France	Co-investigator
Julien Dumurgier	MD, PhD	Memory Resource and Research Centre of Paris Nord, AP-HP, Paris, France	Co-investigator
Diane Dupuy	MD, PhD	Memory Resource and Research of Amiens, CHU Amiens Picardie, F-80000, Amiens, France	Co-investigator
Emmanuelle Duron	MD, PhD	Memory Resource and Research Centre of Paris Broca, AP-HP, Paris, France	Co-investigator
Inna Dygai-Cochet	MD, PhD	Memory Resource and Research Centre of Dijon, CLCC Georges François Leclerc, Dijon, France	Co-investigator
Véronique Eder	MD, PhD	Memory Clinic, Hôpital Avicenne, AP-HP, Hôpitaux Universitaires Paris-Seine-Saint-Denis, F-93009, Bobigny, France	Co-investigator
Stéphane Epelbaum	MD, PhD	Institute of Memory and Alzheimer's Disease (IM2A), Brain and Spine Institute (ICM), UMR S 1127, Department of Neurology, AP-HP, Pitié-Salpêtrière University Hospital, Sorbonne Universities, Pierre et Marie Curie University, Paris, France	Co-investigator
Frédérique Etcharry-Bouyx	MD, PhD	Memory Resource and Research Centre of Angers, CHU d’Angers, F-49000, Angers	Co-investigator
Daniel Fagret	MD, PhD	Memory Resource and Research Centre of Grenoble, CHU de Grenoble Alpes, Grenoble, France	Co-investigator
Catherine Faisant	MD	Memory Resource and Research Centre of Toulouse, CHU de Toulouse, Hôpital La Grave-Casselardit, F-31000, Toulouse, France	Co-investigator
Karim Farid	MD, PhD	Memory Resource and Research Centre of Paris Nord, AP-HP, Paris, France	Co-investigator
Denis Fédérico	MD	Memory Resource and Research Centre of Lyon, Hospices Civils de Lyon, Hôpital des Charpennes, F-69000, Lyon, France	Co-investigator
Olivier Felician	MD, PhD	Memory Resource and Research Centre of Marseille, CHU de Marseille, Hôpital La Timone, F-13000, Marseille, France	Co-investigator
Philippe Fernandez	MD, PhD	Memory Resource and Research Centre of Bordeaux, CHU de Bordeaux, Hôpital Pellegrin, F-33000, Bordeaux, France	Co-investigator
Pacôme Fosse	MD	Memory Resource and Research Centre of Angers, CHU d’Angers, F-49000, Angers	Co-investigator
Alexandra Foubert-Samier	MD, PhD	Memory Resource and Research Centre of Bordeaux, CHU de Bordeaux, Hôpital Pellegrin, F-33000, Bordeaux, France	Co-investigator
Isabelle Franck	MD	Memory Resource and Research Centre of Strasbourg, Hôpitaux Universitaires de Strasbourg, F-67000, Strasbourg, France	Co-investigator
Monique Galitzky	MD	Memory Resource and Research Centre of Toulouse, CHU de Toulouse, Hôpital Purpan, F-31000, Toulouse, France	Co-investigator
Céline Gallazzini-Crepin	MD	Memory Resource and Research Centre of Grenoble, CHU de Grenoble Alpes, Grenoble, France	Co-investigator
Radka Gantchev	MD	Memory Resource and Research Centre of Marseille, CHU de Marseille, Hôpital La Timone, F-13000, Marseille, France	Co-investigator
Laurence Garbarg-Chenon	MD	Memory Clinic, Hôpital Avicenne, AP-HP, Hôpitaux Universitaires Paris-Seine-Saint-Denis, F-93009, Bobigny, France	Co-investigator
Guillaume Gautier	MD, PhD	Memory Resource and Research Centre of Marseille, CHU de Marseille, Hôpital La Timone, F-13000, Marseille, France	Co-investigator
Emmanuel Gerardin	MD, PhD	Memory Resource and Research Centre of Rouen, Neuroradiology Department, Rouen University Hospital, F-76031, Rouen, France	Co-investigator
Claire Gervais	MD	Memory Resource and Research Centre of Nice, CHU de Nice, Institut Claude Pompidou, F-06100, Nice, France	Co-investigator
Jean-Claude Getenet	MD	Memory Resource and Research Centre of Saint-Etienne, CHU de Saint-Etienne, Hôpital Nord, F-42000, Saint-Etienne, France	Co-investigator
Nadine Girard	MD, PhD	Memory Resource and Research Centre of Marseille, CHU de Marseille, Hôpital La Timone, F-13000, Marseille, France	Co-investigator
Fabienne Giraud	MD	Memory Resource and Research Centre of Marseille, CHU de Marseille, Hôpital La Timone, F-13000, Marseille, France	Co-investigator
Chantal Girtanner	MD	Memory Resource and Research Centre of Saint-Etienne, CHU de Saint-Etienne, Hôpital de la Charité, F-42000, Saint-Etienne, France	Co-investigator
Valérie Gissot	MD	Memory Resource and Research Centre of Center Region, CHRU de Tours, Hôpital Bretonneau, F-37000, Tours, France	Co-investigator
Caroline Grangeon	PharmD	Memory Resource and Research Centre of Nice, CHU de Nice, Institut Claude Pompidou, F-06100, Nice, France	Co-investigator
Daniel Grucker	MD, PhD	Memory Resource and Research Centre of Strasbourg, Hôpitaux Universitaires de Strasbourg, F-67000, Strasbourg, France	Co-investigator
Eric Guedj	MD, PhD	Memory Resource and Research Centre of Marseille, CHU de Marseille, Hôpital La Timone, F-13000, Marseille, France	Co-investigator
Claude Gueriot	MD	Memory Resource and Research Centre of Marseille, CHU de Marseille, Hôpital La Timone, F-13000, Marseille, France	Co-investigator
Yves Guilhermet	MD	Memory Resource and Research Centre of Lyon, Hospices Civils de Lyon, Hôpital des Charpennes, F-69000, Lyon, France	Co-investigator
Rémy Guillevin	MD, PhD	Memory Resource and Research Centre of Poitiers, CHU de Poitiers, Hôpital de La Milétrie, F-86000, Poitiers, France	Co-investigator
Sophie Haffen	MD	Memory Resource and Research Centre of Besançon, CHU de Besançon, Hôpital Jean Minjoz, Hôpital Saint-Jacques, F-25000, Besançon, France	Co-investigator
Didier Hannequin	MD, PhD	Memory Resource and Research Centre of Rouen, Neurology Department, Rouen University Hospital, F-76031, Rouen, France	Co-investigator
Sandrine Harston	MD	Memory Resource and Research Centre of Bordeaux, CHU de Bordeaux, Hôpital Xavier Arnozan, F-33000, Bordeaux, France	Co-investigator
Anne Hitzel	MD, PhD	Memory Resource and Research Centre of Toulouse, CHU de Toulouse, Hôpital Purpan, F-31000, Toulouse, France	Co-investigator
Caroline Hommet	MD, PhD	Memory Resource and Research Centre of Center Region, CHRU de Tours, Hôpital Bretonneau, F-37000, Tours, France	Co-investigator
Claude Hossein-Foucher	MD, PhD	Memory Resource and Research Centre of Lille, CHRU de Lille, Hôpital Roger Salengro, F-59000, Lille, France	Co-investigator
Fabrice Hubele	MD	Memory Resource and Research Centre of Strasbourg, Hôpitaux Universitaires de Strasbourg, F-67000, Strasbourg, France	Co-investigator
Agnès Jacquin-Piques	MD, PhD	Memory Resource and Research Centre of Dijon, CHU Dijon Bourgogne, Hôpital du Bocage, Hôpital de Champmaillot, F-21000, Dijon, France	Co-investigator
Betty Jean	MD	Memory Resource and Research Centre of Clermont-Ferrand, CHU de Clermont-Ferrand, F-63000, Clermont-Ferrand, France	Co-investigator
Joanne Jenn	MD, PhD	Memory Resource and Research Centre of Bordeaux, CHU de Bordeaux, Hôpital Xavier Arnozan, F-33000, Bordeaux, France	Co-investigator
Laure Joly	MD, PhD	Memory Resource and Research Centre of Nancy, CHU de Nancy, F-54000, Nancy, France	Co-investigator
Thérèse Jonveaux	MD	Memory Resource and Research Centre of Nancy, CHU de Nancy, F-54000, Nancy, France	Co-investigator
Adrien Julian	MD, PhD	Memory Resource and Research Centre of Poitiers, CHU de Poitiers, Hôpital de La Milétrie, F-86000, Poitiers, France	Co-investigator
Aurélie Kas	MD, PhD	Laboratoire d'Imagerie Biomédicale, Sorbonne Universités, UPMC Univ Paris 06, Inserm U1146, CNRS UMR 7371, France NeuroSpin, I2BM, Commissariat à l'Energie Atomique, Paris, France	Co-investigator
Anna Kearney-Schwartz	MD	Memory Resource and Research Centre of Nancy, CHU de Nancy, F-54000, Nancy, France	Co-investigator
Alice Keles	MD	Memory Resource and Research Centre of Nancy, CHU de Nancy, F-54000, Nancy, France	Co-investigator
Antony Kelly	MD	Memory Resource and Research Centre of Clermont-Ferrand, Centre de Lutte contre le Cancer, F-63000, Clermont-Ferrand, France	Co-investigator
Nathalie Keromnes	MD	Memory Resource and Research Centre of Brest, CHRU de Brest, F-29000, Brest, France	Co-investigator
Lejla Koric	MD	Memory Resource and Research Centre of Marseille, CHU de Marseille, Hôpital La Timone, F-13000, Marseille, France	Co-investigator
Alexandre Krainik	MD, PhD	Memory Resource and Research Centre of Grenoble, CHU de Grenoble Alpes, Grenoble, France	Co-investigator
Stéphane Kremer	MD	Memory Resource and Research Centre of Strasbourg, Hôpitaux Universitaires de Strasbourg, F-67000, Strasbourg, France	Co-investigator
Florian Labourée	MD	Memory Resource and Research Centre of Paris Broca, AP-HP, Paris, France	Co-investigator
Franck Lacoeuille	MD, PhD	Memory Resource and Research Centre of Angers, CHU d’Angers, F-49000, Angers	Co-investigator
Francoise Lala	MD	Memory Resource and Research Centre of Toulouse, CHU de Toulouse, Hôpital La Grave-Casselardit, F-31000, Toulouse, France	Co-investigator
Chantal Lamy	MD	Memory Resource and Research of Amiens, CHU Amiens Picardie, F-80000, Amiens, France	Co-investigator
Jean-Louis Laplanche	PharmD, PhD	Memory Resource and Research Centre of Paris Nord, AP-HP, Paris, France	Co-investigator
Cyrille Launay	MD, PhD	Memory Resource and Research Centre of Angers, CHU d’Angers, F-49000, Angers	Co-investigator
Stéphane Lehericy	MD, PhD	Institute of Memory and Alzheimer's Disease (IM2A), Centre for NeuroImaging Research (CENIR), Brain and Spine Institute (ICM), UMR S 1127, Department of Neurology, AP-HP, Pitié-Salpêtrière University Hospital, Sorbonne Universities, Pierre et Marie Curie University, Paris, France	Co-investigator
Sylvain Lehmann	MD, PhD	Memory Resource and Research Centre of Montpellier, CHU de Montpellier, Hôpital Gui de Chauliac, F-34000, Montpellier, France	Co-investigator
Hermine Lenoir	MD, PhD	Memory Resource and Research Centre of Paris Broca, AP-HP, Paris, France	Co-investigator
Marcel Levy	MD, PhD	Institute of Memory and Alzheimer's Disease (IM2A), Brain and Spine Institute (ICM), UMR S 1127, Department of Neurology, AP-HP, Pitié-Salpêtrière University Hospital, Sorbonne Universities, Pierre et Marie Curie University, Paris, France	Co-investigator
Stéphanie Libercier	MD, PhD	Memory Resource and Research Centre of Colmar, Hôpitaux Civils de Colmar, F-68000, Colmar, France	Co-investigator
Marie-Anne Mackowiak-Cordoliani	MD	Memory Resource and Research Centre of Lille, CHRU de Lille, Hôpital Roger Salengro, F-59000, Lille, France	Co-investigator
Eloi Magnin	MD	Memory Resource and Research Centre of Besançon, CHU de Besançon, Hôpital Jean Minjoz, Hôpital Saint-Jacques, F-25000, Besançon, France	Co-investigator
Zaza Makaroff	MD	Memory Resource and Research Centre of Lyon, Hospices Civils de Lyon, Hôpital des Charpennes, F-69000, Lyon, France	Co-investigator
Athina Marantidou	MD	Memory Clinic, Hôpital Avicenne, AP-HP, Hôpitaux Universitaires Paris-Seine-Saint-Denis, F-93009, Bobigny, France	Co-investigator
Isabelle Marcet	MD	Memory Resource and Research Centre of Bordeaux, CHU de Bordeaux, Hôpital Pellegrin, F-33000, Bordeaux, France	Co-investigator
Cécilia Marelli	MD, PhD	Memory Resource and Research Centre of Montpellier, CHU de Montpellier, Hôpital Gui de Chauliac, F-34000, Montpellier, France	Co-investigator
Sophie Marilier	MD	Memory Resource and Research Centre of Dijon, CHU Dijon Bourgogne, Hôpital du Bocage, Hôpital de Champmaillot, F-21000, Dijon, France	Co-investigator
Idalie Martin	MD	Memory Resource and Research Centre of Lyon, Hospices Civils de Lyon, Hôpital des Charpennes, F-69000, Lyon, France	Co-investigator
Olivier Martinaud	MD, PhD	Memory Resource and Research Centre of Rouen, Neurology Department, Rouen University Hospital, F-76031, Rouen, France	Co-investigator
Catherine Martin-Hunyadi	MD	Memory Resource and Research Centre of Strasbourg, Hôpitaux Universitaires de Strasbourg, F-67000, Strasbourg, France	Co-investigator
Aïcha Medjoul	MD	Memory Clinic, Hôpital Avicenne, AP-HP, Hôpitaux Universitaires Paris-Seine-Saint-Denis, F-93009, Bobigny, France	Co-investigator
Isabelle Merlet	MD	Memory Resource and Research Centre of Poitiers, CHU de Poitiers, Hôpital de La Milétrie, F-86000, Poitiers, France	Co-investigator
Danielle Mestas	MD	Memory Resource and Research Centre of Clermont-Ferrand, CHU de Clermont-Ferrand, F-63000, Clermont-Ferrand, France	Co-investigator
Marc-Etienne Meyer	MD, PhD	Memory Resource and Research of Amiens, CHU Amiens Picardie, F-80000, Amiens, France	Co-investigator
Jean-Marc Michel	MD	Memory Resource and Research Centre of Colmar, Hôpitaux Civils de Colmar, F-68000, Colmar, France	Co-investigator
Agnès Michon	MD	Institute of Memory and Alzheimer's Disease (IM2A), Brain and Spine Institute (ICM), UMR S 1127, Department of Neurology, AP-HP, Pitié-Salpêtrière University Hospital, Sorbonne Universities, Pierre et Marie Curie University, Paris, France	Co-investigator
Isabelle Migeon-Duballet	MD	Memory Resource and Research Centre of Poitiers, CHU de Poitiers, Hôpital de La Milétrie, F-86000, Poitiers, France	Co-investigator
Karl Mondon	MD, PhD	Memory Resource and Research Centre of Center Region, CHRU de Tours, Hôpital Bretonneau, F-37000, Tours, France	Co-investigator
Clément Morgat	PharmD, PhD	Memory Resource and Research Centre of Bordeaux, CHU de Bordeaux, Hôpital Pellegrin, F-33000, Bordeaux, France	Co-investigator
Véronique Moullart	MD	Memory Resource and Research of Amiens, CHU Amiens Picardie, F-80000, Amiens, France	Co-investigator
Christian Moussard	MD	Memory Resource and Research Centre of Besançon, CHU de Besançon, Hôpital Jean Minjoz, Hôpital Saint-Jacques, F-25000, Besançon, France	Co-investigator
Aurélie Mouton	MD, PhD	Memory Resource and Research Centre of Nice, CHU de Nice, Institut Claude Pompidou, F-06100, Nice, France	Co-investigator
Izzie Jacques Namer	MD, PhD	Memory Resource and Research Centre of Strasbourg, Hôpitaux Universitaires de Strasbourg, F-67000, Strasbourg, France	Co-investigator
Georges Niewiadomski	MD, PhD	Memory Resource and Research Centre of Nice, CHU de Nice, Institut Claude Pompidou, F-06100, Nice, France	Co-investigator
Guillaume Nivaggioni	MD	Memory Resource and Research Centre of Nice, CHU de Nice, Institut Claude Pompidou, F-06100, Nice, France	Co-investigator
Marie Noblet	MD, PhD	Memory Resource and Research Centre of Strasbourg, Hôpitaux Universitaires de Strasbourg, F-67000, Strasbourg, France	Co-investigator
Michel Nonent	MD, PhD	Memory Resource and Research Centre of Brest, CHRU de Brest, F-29000, Brest, France	Co-investigator
Fati Nourhashemi	MD, PhD	Memory Resource and Research Centre of Toulouse, CHU de Toulouse, Hôpital La Grave-Casselardit, F-31000, Toulouse, France	Co-investigator
Hélène Oesterle	MD	Memory Resource and Research Centre of Colmar, Hôpitaux Civils de Colmar, F-68000, Colmar, France	Co-investigator
Galdric Orvoen	MD	Memory Resource and Research Centre of Paris Broca, AP-HP, Paris, France	Co-investigator
Pierre Jean Ousset	MD, PhD	Memory Resource and Research Centre of Toulouse, CHU de Toulouse, Hôpital La Grave-Casselardit, F-31000, Toulouse, France	Co-investigator
Amandine Pallardy	MD	Memory Resource and Research Centre of Nantes, CHU de Nantes, F-44000, Nantes, France	Co-investigator
Claire Paquet	MD, PhD	Memory Resource and Research Centre of Paris Nord, AP-HP, Paris, France	Co-investigator
Pierre-Yves Pare	MD, PhD	Memory Resource and Research Centre of Angers, CHU d’Angers, F-49000, Angers	Co-investigator
Anne Pasco	MD, PhD	Memory Resource and Research Centre of Angers, CHU d’Angers, F-49000, Angers	Co-investigator
Pierre Payoux	MD, PhD	Memory Resource and Research Centre of Toulouse, CHU de Toulouse, Hôpital Purpan, F-31000, Toulouse, France	Co-investigator
Cécile Pays	MD, PhD	Memory Resource and Research Centre of Montpellier, CHU de Montpellier, Hôpital Gui de Chauliac, F-34000, Montpellier, France	Co-investigator
Isabelle Pellegrin	MD, PhD	Biological Research Centre, CHU de Bordeaux, F-33000, Bordeaux, France	Co-investigator
Rémy Perdrisot	MD, PhD	Memory Resource and Research Centre of Poitiers, CHU de Poitiers, Hôpital de La Milétrie, F-86000, Poitiers, France	Co-investigator
Bertille Perin	MD, PhD	Memory Resource and Research of Amiens, CHU Amiens Picardie, F-80000, Amiens, France	Co-investigator
Christine Perret-Guillaume	MD, PhD	Memory Resource and Research Centre of Nancy, CHU de Nancy, F-54000, Nancy, France	Co-investigator
Grégory Petyt	MD	Memory Resource and Research Centre of Lille, CHRU de Lille, Hôpital Roger Salengro, F-59000, Lille, France	Co-investigator
Nathalie Philippi	MD, PhD	Memory Resource and Research Centre of Strasbourg, Hôpitaux Universitaires de Strasbourg, F-67000, Strasbourg, France	Co-investigator
Geneviève Pinganaud	MD	Memory Resource and Research Centre of Bordeaux, CHU de Bordeaux, Hôpital Xavier Arnozan, F-33000, Bordeaux, France	Co-investigator
Matthieu Plichart	MD	Memory Resource and Research Centre of Paris Broca, AP-HP, Paris, France	Co-investigator
Gabriel Pop	MD, PhD	Memory Clinic, Hôpital Avicenne, AP-HP, Hôpitaux Universitaires Paris-Seine-Saint-Denis, F-93009, Bobigny, France	Co-investigator
Michèle Puel	MD	Memory Resource and Research Centre of Toulouse, CHU de Toulouse, Hôpital Purpan, F-31000, Toulouse, France	Co-investigator
Mathieu Queneau	MD, PhD	Memory Resource and Research Centre of Paris Nord, Centre Cardiologique du Nord, Paris, France	Co-investigator
Solène Querellou	MD	Memory Resource and Research Centre of Brest, CHRU de Brest, F-29000, Brest, France	Co-investigator
Muriel Quillard-Muraine	MD, PhD	Memory Resource and Research Centre of Rouen, Neurology Department, Rouen University Hospital, F-76031, Rouen, France	Co-investigator
Valérie Quipourt	MD, PhD	Memory Resource and Research Centre of Dijon, CHU Dijon Bourgogne, Hôpital du Bocage, Hôpital de Champmaillot, F-21000, Dijon, France	Co-investigator
Chloé Rachez	MD, PhD	Memory Resource and Research Centre of Clermont-Ferrand, CHU de Clermont-Ferrand, F-63000, Clermont-Ferrand, France	Co-investigator
Micheline Razzouk-Cadet	MD	Memory Resource and Research Centre of Nice, CHU de Nice, Institut Claude Pompidou, F-06100, Nice, France	Co-investigator
Anne-Sophie Rigaud	MD, PhD	Memory Resource and Research Centre of Paris Broca, AP-HP, Paris, France	Co-investigator
Hélène Robin-Ismer	MD	Memory Resource and Research Centre of Strasbourg, Hôpitaux Universitaires de Strasbourg, F-67000, Strasbourg, France	Co-investigator
Mathieu Rodallec	MD, PhD	Memory Resource and Research Centre of Paris Nord, Centre Cardiologique du Nord, Paris, France	Co-investigator
Yves Rolland	MD, PhD	Memory Resource and Research Centre of Toulouse, CHU de Toulouse, Hôpital La Grave-Casselardit, F-31000, Toulouse, France	Co-investigator
Adeline Rollin-Sillaire	MD, PhD	Memory Resource and Research Centre of Lille, CHRU de Lille, Hôpital Roger Salengro, F-59000, Lille, France	Co-investigator
Olivier Rouaud	MD	Memory Resource and Research Centre of Dijon, CHU Dijon Bourgogne, Hôpital du Bocage, Hôpital de Champmaillot, F-21000, Dijon, France	Co-investigator
Caroline Roubaud	MD, PhD	Memory Resource and Research Centre of Lyon, Hospices Civils de Lyon, Hôpital des Charpennes, F-69000, Lyon, France	Co-investigator
Isabelle Rouch	MD, PhD	Memory Resource and Research Centre of Lyon, Hospices Civils de Lyon, Hôpital des Charpennes, F-69000, Lyon, France	Co-investigator
Julie Roux	MD, PhD	Memory Resource and Research Centre of Grenoble, CHU de Grenoble Alpes, Grenoble, France	Co-investigator
Guillaume Sacco	MD, PhD	Memory Resource and Research Centre of Nice, CHU de Nice, Institut Claude Pompidou, F-06100, Nice, France	Co-investigator
Pierre-Yves Salaun	MD	Memory Resource and Research Centre of Brest, CHRU de Brest, F-29000, Brest, France	Co-investigator
François Salmon	MD, PhD	Memory Resource and Research Centre of Poitiers, CHU de Poitiers, Hôpital de La Milétrie, F-86000, Poitiers, France	Co-investigator
Alicia Sanchez	MD	Memory Resource and Research Centre of Saint-Etienne, CHU de Saint-Etienne, Hôpital Nord, F-42000, Saint-Etienne, France	Co-investigator
Maria-Joao Santiago-Ribeiro	MD, PhD	Memory Resource and Research Centre of Center Region, CHRU de Tours, Hôpital Bretonneau, F-37000, Tours, France	Co-investigator
Alain Sarciron	MD	Memory Resource and Research Centre of Lyon, Hospices Civils de Lyon, Hôpital des Charpennes, F-69000, Lyon, France	Co-investigator
Nathalie Sastre-Hengan	MD	Memory Resource and Research Centre of Toulouse, CHU de Toulouse, Hôpital La Grave-Casselardit, F-31000, Toulouse, France	Co-investigator
Mathilde Sauvée	MD, PhD	Memory Resource and Research Centre of Grenoble, CHU de Grenoble Alpes, Grenoble, France	Co-investigator
Christian Scheiber	MD, PhD	Memory Resource and Research Centre of Lyon, Hospices Civils de Lyon, Hôpital des Charpennes, F-69000, Lyon, France	Co-investigator
Anne-Marie Schneider	MD, PhD	Memory Resource and Research Centre of Strasbourg, Hôpitaux Universitaires de Strasbourg, F-67000, Strasbourg, France	Co-investigator
Franck Semah	MD, PhD	Memory Resource and Research Centre of Lille, CHRU de Lille, Hôpital Roger Salengro, F-59000, Lille, France	Co-investigator
Amélie Serra	MD	Memory Resource and Research Centre of Grenoble, CHU de Grenoble Alpes, Grenoble, France	Co-investigator
Marie-Laure Seux	MD	Memory Resource and Research Centre of Paris Broca, AP-HP, Paris, France	Co-investigator
Hélène Sordet-Guépet	MD	Memory Resource and Research Centre of Dijon, CHU Dijon Bourgogne, Hôpital du Bocage, Hôpital de Champmaillot, F-21000, Dijon, France	Co-investigator
Maria Eugenia Soto	MD	Memory Resource and Research Centre of Toulouse, CHU de Toulouse, Hôpital La Grave-Casselardit, F-31000, Toulouse, France	Co-investigator
Mathieu Tafani	MD	Memory Resource and Research Centre of Toulouse, CHU de Toulouse, Hôpital Purpan, F-31000, Toulouse, France	Co-investigator
Jean-Yves Tanguy	MD, PhD	Memory Resource and Research Centre of Angers, CHU d’Angers, F-49000, Angers	Co-investigator
Michael Taroux	MD, PhD	Memory Resource and Research Centre of Dijon, CHU Dijon Bourgogne, Hôpital du Bocage, Hôpital de Champmaillot, F-21000, Dijon, France	Co-investigator
Marc Teichmann	MD, PhD	Institute of Memory and Alzheimer's Disease (IM2A), Brain and Spine Institute (ICM), UMR S 1127, Department of Neurology, AP-HP, Pitié-Salpêtrière University Hospital, Sorbonne Universities, Pierre et Marie Curie University, Paris, France	Co-investigator
Catherine Terrat	MD, PhD	Memory Resource and Research Centre of Saint-Etienne, CHU de Saint-Etienne, Hôpital de la Charité, F-42000, Saint-Etienne, France	Co-investigator
Jamila Thabet	MD	Memory Clinic, Hôpital Avicenne, AP-HP, Hôpitaux Universitaires Paris-Seine-Saint-Denis, F-93009, Bobigny, France	Co-investigator
Claire Thalamas	MD	Memory Resource and Research Centre of Toulouse, CHU de Toulouse, Hôpital Purpan, F-31000, Toulouse, France	Co-investigator
Catherine Thomas-Anterion	MD, PhD	Memory Resource and Research Centre of Saint-Etienne, CHU de Saint-Etienne, Hôpital Nord, F-42000, Saint-Etienne, France	Co-investigator
Anne-Cécile Troussière	MD	Memory Resource and Research Centre of Lille, CHRU de Lille, Hôpital Roger Salengro, F-59000, Lille, France	Co-investigator
Renata Ursu	MD	Memory Clinic, Hôpital Avicenne, AP-HP, Hôpitaux Universitaires Paris-Seine-Saint-Denis, F-93009, Bobigny, France	Co-investigator
Pierre Vera	MD, PhD	Memory Resource and Research Centre of Rouen, CLCC Henri Becquerel, Rouen, France	Co-investigator
Martine Vercelletto	MD	Memory Resource and Research Centre of Nantes, CHU de Nantes, F-44000, Nantes, France	Co-investigator
Olivier Vercruysse	MD	Memory Resource and Research Centre of Lille, CHRU de Lille, Hôpital Roger Salengro, F-59000, Lille, France	Co-investigator
Antoine Verger	MD, PhD	Memory Resource and Research Centre of Nancy, CHU de Nancy, F-54000, Nancy, France	Co-investigator
Philippe Viau	MD	Memory Resource and Research Centre of Nice, CHU de Nice, Institut Claude Pompidou, F-06100, Nice, France	Co-investigator
Marie-Neige Videau	MD	Memory Resource and Research Centre of Bordeaux, CHU de Bordeaux, Hôpital Xavier Arnozan, F-33000, Bordeaux, France	Co-investigator
Thierry Voisin	MD	Memory Resource and Research Centre of Toulouse, CHU de Toulouse, Hôpital La Grave-Casselardit, F-31000, Toulouse, France	Co-investigator
Nathalie Wagemann	MD, PhD	Memory Resource and Research Centre of Nantes, CHU de Nantes, F-44000, Nantes, France	Co-investigator
Aziza Waissi-Sediq	MD	Memory Resource and Research Centre of Lyon, Hospices Civils de Lyon, Hôpital des Charpennes, F-69000, Lyon, France	Co-investigator
Jing Xie	MD, PhD	Memory Resource and Research Centre of Lyon, Hospices Civils de Lyon, Hôpital des Charpennes, F-69000, Lyon, France	Co-investigator
Nathanaëlle Yeni	MD	Laboratoire d'Imagerie Biomédicale, Sorbonne Universités, UPMC Univ Paris 06, Inserm U1146, CNRS UMR 7371, France NeuroSpin, I2BM, Commissariat à l'Energie Atomique, Paris, France	Co-investigator
Michel Zanca	MD, PhD	Memory Resource and Research Centre of Montpellier, CHU de Montpellier, Hôpital Gui de Chauliac, F-34000, Montpellier, France	Co-investigator
Jean Zinszner	MD, PhD	Memory Clinic, Hôpital Avicenne, AP-HP, Hôpitaux Universitaires Paris-Seine-Saint-Denis, F-93009, Bobigny, France	Co-investigator

## Abbreviations

AD: Alzheimer disease
CSF: Cerebrospinal fluid
ATN: Amyloid Tau Neurodegeneration
PET: Positron emission tomography
MCI: Mild cognitive impairment
NPS: Neuropsychiatric symptoms
MBI: Mild behavioral impairment
NPSnotMBI: NPS not meeting MBI criteria
CN: Cognitively normal / normal cognition
MMSE: Mini Mental State Exam
LME: Linear mixed effects
NPI: Neuropsychiatric Inventory
Aβ: myloid beta
p-tau: hyperphosporylated tau
t-tau: total tau

## References

[1] Gauthier S, Albert M, Fox N, Goedert M, Kivipelto M, Mestre-Ferrandiz J (2016). Why has therapy development for dementia failed in the last two decades?. Alzheimers Dement.

[2] Ferreira D, Rivero-Santana A, Perestelo-Pérez L, Westman E, Wahlund L-O, Sarría A (2014). Improving CSF biomarkers’ performance for predicting progression from mild cognitive impairment to Alzheimer’s disease by considering different confounding factors: a meta-analysis. Front Aging Neurosci.

[3] Ruan Q, D’Onofrio G, Sancarlo D, Bao Z, Greco A, Yu Z (2016). Potential neuroimaging biomarkers of pathologic brain changes in Mild Cognitive Impairment and Alzheimer’s disease: a systematic review. BMC Geriatr.

[4] Dunne RA, Aarsland D, O’Brien JT, Ballard C, Banerjee S, Fox NC (2021). Mild cognitive impairment: the Manchester consensus. Age Ageing.

[5] Belleville S, Fouquet C, Hudon C, Zomahoun HTV, Croteau J (2017). Neuropsychological measures that predict progression from mild cognitive impairment to Alzheimer's type dementia in older adults: a systematic review and meta-analysis. Neuropsychol Rev.

[6] Lombardi G, Crescioli G, Cavedo E, Lucenteforte E, Casazza G, Bellatorre AG, et al. Structural magnetic resonance imaging for the early diagnosis of dementia due to Alzheimer's disease in people with mild cognitive impairment. Cochrane Database Syst Rev. 2020(3). 10.1002/14651858.CD009628.pub2 PMC7059964 32119112

[7] Watermeyer T, Calia C. Neuropsychological assessment in preclinical and prodromal Alzheimer disease: a global perspective. J Global Health. 2019;9(1). 10.7189/jogh.09.010317 PMC6486120 31073397

[8] Wise EA, Rosenberg PB, Lyketsos CG, Leoutsakos J-M (2019). Time course of neuropsychiatric symptoms and cognitive diagnosis in National Alzheimer's Coordinating Centers volunteers. Alzheimers Dement (Amst).

[9] Taragano FE, Allegri RF, Krupitzki H, Sarasola D, Serrano C, Lyketsos C (2009). Mild behavioral impairment. J Clin Psychiatry.

[10] Ismail Z, Smith EE, Geda Y, Sultzer D, Brodaty H, Smith G (2016). Neuropsychiatric symptoms as early manifestations of emergent dementia: provisional diagnostic criteria for mild behavioral impairment. Alzheimers Dement.

[11] Jack CR, Bennett DA, Blennow K, Carrillo MC, Dunn B, Haeberlein SB (2018). NIA-AA research framework: toward a biological definition of Alzheimer's disease. Alzheimers Dement.

[12] Ismail Z, McGirr A, Gill S, Hu S, Forkert ND, Smith EE (2021). Mild Behavioral Impairment and Subjective Cognitive Decline Predict Cognitive and Functional Decline. J Alzheimers Dis.

[13] McGirr A, Nathan S, Ghahremani M, Gill S, Smith EE, Ismail Z (2022). Progression to Dementia or Reversion to Normal Cognition in Mild Cognitive Impairment as a Function of Late-Onset Neuropsychiatric Symptoms. Neurology.

[14] Creese B, Brooker H, Ismail Z, Wesnes KA, Hampshire A, Khan Z (2019). Mild Behavioral Impairment as a Marker of Cognitive Decline in Cognitively Normal Older Adults. Am J Geriatr Psychiatry.

[15] Kan CN, Cano J, Zhao X, Ismail Z, Chen CL, Xu X (2022). Prevalence, Clinical Correlates, Cognitive Trajectories, and Dementia Risk Associated With Mild Behavioral Impairment in Asians. J Clin Psychiatry.

[16] Matsuoka T, Ismail Z, Narumoto J (2019). Prevalence of mild behavioral impairment and risk of dementia in a psychiatric outpatient clinic. J Alzheimers Dis.

[17] Ruthirakuhan M, Ismail Z, Herrmann N, Gallagher D, Lanctot KL. Mild behavioral impairment is associated with progression to Alzheimer's disease: A clinicopathological study. Alzheimers Dement. 2022;in press. 10.1002/alz.12519 PMC9339594 35103400

[18] Tsunoda K, Yamashita T, Osakada Y, Sasaki R, Tadokoro K, Matsumoto N (2021). Positive baseline behavioral and psychological symptoms of dementia predict a subsequent cognitive impairment in cognitively normal population. Neurol Clin Neurosci.

[19] Wolfova K, Creese B, Aarsland D, Ismail Z, Corbett A, Ballard C (2022). Gender/Sex Differences in the Association of Mild Behavioral Impairment with Cognitive Aging. J Alzheimers Dis.

[20] Gill S, Mouches P, Hu S, Rajashekar D, MacMaster FP, Smith EE (2020). Using Machine Learning to Predict Dementia from Neuropsychiatric Symptom and Neuroimaging Data. J Alzheimers Dis.

[21] Ebrahim IM, Ghahremani M, Camicioli R, Smith EE, Ismail Z (2023). Effects of race, baseline cognition, and APOE on the association of affective dysregulation with incident dementia: A longitudinal study of dementia-free older adults. J Affect Disord.

[22] Ismail Z, Ghahremani M, Amlish Munir M, Fischer CE, Smith EE, Creese B (2023). A longitudinal study of late-life psychosis and incident dementia and the potential effects of race and cognition. Nat Mental Health.

[23] Yoon EJ, Lee J-Y, Kwak S, Kim YK. Mild behavioral impairment linked to progression to Alzheimer’s disease and cortical thinning in amnestic mild cognitive impairment. Front Aging Neurosci. 2023;14. 10.3389/fnagi.2022.1051621 PMC9846631 36688162

[24] Rouse HJ, Ismail Z, Andel R, Molinari VA, Schinka JA, Small BJ. Impact of Mild Behavioral Impairment on Longitudinal Changes in Cognition. J Gerontol: Series A. 2023:glad098. 10.1093/gerona/glad098 PMC11491738 37052173

[25] Creese B, Arathimos R, Aarsland D, Ballard C, Brooker H, Hampshire A (2023). Late-life onset psychotic symptoms and incident cognitive impairment in people without dementia: Modification by genetic risk for Alzheimer's disease. Alzheimer's & Dementia: Transl Res & Clin Intervent.

[26] Johansson M, Stomrud E, Insel PS, Leuzy A, Johansson PM, Smith R (2021). Mild behavioral impairment and its relation to tau pathology in preclinical Alzheimer's disease. Transl Psychiatr.

[27] Lussier FZ, Pascoal TA, Chamoun M, Therriault J, Tissot C, Savard M (2020). Mild behavioral impairment is associated with β-amyloid but not tau or neurodegeneration in cognitively intact elderly individuals. Alzheimers Dement.

[28] Miao R, Chen HY, Gill S, Naude J, Smith EE, Ismail Z (2022). Plasma beta-Amyloid in Mild Behavioural Impairment - Neuropsychiatric Symptoms on the Alzheimer's Continuum. J Geriatr Psychiatry Neurol.

[29] Dufouil C, Dubois B, Vellas B, Pasquier F, Blanc F, Hugon J (2017). Cognitive and imaging markers in non-demented subjects attending a memory clinic: study design and baseline findings of the MEMENTO cohort. Alzheimers Res Ther.

[30] Cummings J (2020). The Neuropsychiatric Inventory: Development and Applications. J Geriatr Psychiatry Neurol.

[31] Sheikh F, Ismail Z, Mortby ME, Barber P, Cieslak A, Fischer K (2018). Prevalence of mild behavioral impairment in mild cognitive impairment and subjective cognitive decline, and its association with caregiver burden. Int Psychogeriatr.

[32] Howard AL (2015). Leveraging time-varying covariates to test within-and between-person effects and interactions in the multilevel linear model. Emerg Adulthood.

[33] Moussa C, Hebron M, Huang X, Ahn J, Rissman RA, Aisen PS (2017). Resveratrol regulates neuro-inflammation and induces adaptive immunity in Alzheimer’s disease. J Neuroinflammation.

[34] Noguchi-Shinohara M, Komatsu J, Samuraki M, Matsunari I, Ikeda T, Sakai K (2017). Cerebral amyloid angiopathy-related microbleeds and cerebrospinal fluid biomarkers in Alzheimer’s disease. J Alzheimers Dis.

[35] Hellström-Lindahl E, Mousavi M, Ravid R, Nordberg A (2004). Reduced levels of Aβ 40 and Aβ 42 in brains of smoking controls and Alzheimer's patients. Neurobiol Dis.

[36] Walsh P, Sudre CH, Fiford CM, Ryan NS, Lashley T, Frost C (2021). The age-dependent associations of white matter hyperintensities and neurofilament light in early-and late-stage Alzheimer's disease. Neurobiol Aging.

[37] Miao R, Chen H-Y, Robert P, Smith EE, Ismail Z, Group MS (2021). White matter hyperintensities and mild behavioral impairment: Findings from the MEMENTO cohort study. Cerebral Circulation-Cognition and Behavior.

[38] Fukuyama R, Mizuno T, Mizuno T, Mori S, Nakajima K, Fushiki S (2000). Age-dependent change in the levels of Aβ40 and Aβ42 in cerebrospinal fluid from control subjects, and a decrease in the ratio of Aβ42 to Aβ40 level in cerebrospinal fluid from Alzheimer’s disease patients. Eur Neurol.

[39] Hansson O, Zetterberg H, Buchhave P, Andreasson U, Londos E, Minthon L (2007). Prediction of Alzheimer’s disease using the CSF Aβ42/Aβ40 ratio in patients with mild cognitive impairment. Dement Geriatr Cogn Disord.

[40] Showraki A, Murari G, Ismail Z, Barfett JJ, Fornazzari L, Munoz DG (2019). Cerebrospinal Fluid Correlates of Neuropsychiatric Symptoms in Patients with Alzheimer’s Disease/Mild Cognitive Impairment: A Systematic Review. J Alzheimers Dis.

[41] Ghahremani M, Wang M, Chen H-Y, Zetterberg H, Smith E, Ismail Z (2023). Plasma Phosphorylated Tau at Threonine 181 and Neuropsychiatric Symptoms in Preclinical and Prodromal Alzheimer Disease. Neurology.

[42] Ismail Z, Aguera-Ortiz L, Brodaty H, Cieslak A, Cummings J, Fischer CE (2017). The Mild Behavioral Impairment Checklist (MBI-C): A Rating Scale for Neuropsychiatric Symptoms in Pre-Dementia Populations. J Alzheimers Dis.

[43] Hansson O, Seibyl J, Stomrud E, Zetterberg H, Trojanowski JQ, Bittner T (2018). CSF biomarkers of Alzheimer's disease concord with amyloid-β PET and predict clinical progression: a study of fully automated immunoassays in BioFINDER and ADNI cohorts. Alzheimers Dement.

[44] Santangelo R, Masserini F, Agosta F, Sala A, Caminiti SP, Cecchetti G, et al. CSF p-tau/Aβ 42 ratio and brain FDG-PET may reliably detect MCI “imminent” converters to AD. Eur J Nucl Med Mol Imaging. 2020:1–13. 10.1007/s00259-020-04853-4 32415550

[45] Naude JP, Gill S, Hu S, McGirr A, Forkert ND, Monchi O (2020). Plasma Neurofilament Light: A Marker of Neurodegeneration in Mild Behavioral Impairment. J Alzheimers Dis.

[46] Kassam F, Chen H, Nosheny RL, McGirr A, Williams T, Ng N, et al. Cognitive profile of people with mild behavioral impairment in Brain Health Registry participants. Int Psychogeriatr. 2022;in press:1–10. 10.1017/S1041610221002878 PMC10063171 35130991

[47] Rouse HJ, Small BJ, Schinka JA, Loewenstein DA, Duara R, Potter H (2021). Mild behavioral impairment as a predictor of cognitive functioning in older adults. Int Psychogeriatr.

[48] Andrews SJ, Ismail Z, Anstey KJ, Mortby M (2018). Association of Alzheimer's genetic loci with mild behavioral impairment. Am J Med Genet B Neuropsychiatr Genet.

[49] Creese B, Arathimos R, Brooker H, Aarsland D, Corbett A, Lewis C (2021). Genetic risk for Alzheimer's disease, cognition, and mild behavioral impairment in healthy older adults. Alzheimers Dement (Amst).

[50] Gill S, Wang M, Mouches P, Rajashekar D, Sajobi T, MacMaster FP (2021). Neural correlates of the impulse dyscontrol domain of mild behavioral impairment. Int J Geriatr Psychiatry.

[51] Matuskova V, Ismail Z, Nikolai T, Markova H, Cechova K, Nedelska Z (2021). Mild behavioral impairment is associated with atrophy of entorhinal cortex and hippocampus in a memory clinic cohort. Frontiers in Aging Neuroscience.

[52] Matsuoka T, Imai A, Narumoto J (2023). Neuroimaging of mild behavioral impairment: A systematic review. Psychiatry and Clinical Neurosciences Reports.

[53] Taragano FE, Allegri RF, Heisecke SL, Martelli MI, Feldman ML, Sánchez V (2018). Risk of Conversion to Dementia in a Mild Behavioral Impairment Group Compared to a Psychiatric Group and to a Mild Cognitive Impairment Group. J Alzheimers Dis.

[54] Creavin ST, Wisniewski S, Noel‐Storr AH, Trevelyan CM, Hampton T, Rayment D, et al. Mini‐Mental State Examination (MMSE) for the detection of dementia in clinically unevaluated people aged 65 and over in community and primary care populations. The Cochrane Library. 2016. 10.1002/14651858.CD011145.pub2 PMC8812342 26760674

[55] Creese B, Griffiths A, Brooker H, Corbett A, Aarsland D, Ballard C (2020). Profile of mild behavioral impairment and factor structure of the Mild Behavioral Impairment Checklist in cognitively normal older adults. Int Psychogeriatr.

[56] Cui Y, Dai S, Miao Z, Zhong Y, Liu Y, Liu L (2019). Reliability and Validity of the Chinese Version of the Mild Behavioral Impairment Checklist for Screening for Alzheimer’s Disease. J Alzheimers Dis.

[57] Hu S, Patten S, Charlton A, Fischer K, Fick G, Smith EE, et al. Validating the Mild Behavioral Impairment Checklist in a Cognitive Clinic: Comparisons With the Neuropsychiatric Inventory Questionnaire. J Geriatr Psychiatry Neurol. 2022:8919887221093353. 10.1177/08919887221093353 PMC9941652 35430902

[58] Lin RS, Yu DS, Chau PH, Li PW, Ismail Z. Reliability and Validity of the Traditional Chinese Version of the Mild Behavioral Impairment–Checklist Among Persons With Mild Cognitive Impairment–A Validation Study. J Geriatr Psychiatry Neurol. 2022:08919887221093363. 10.1177/08919887221093363 35430911

[59] Mallo SC, Ismail Z, Pereiro AX, Facal D, Lojo-Seoane C, Campos-Magdaleno M (2018). Assessing mild behavioral impairment with the Mild behavioral impairment-checklist in people with mild cognitive impairment. J Alzheimers Dis.

[60] Mallo SC, Ismail Z, Pereiro AX, Facal D, Lojo-Seoane C, Campos-Magdaleno M (2019). Assessing mild behavioral impairment with the mild behavioral impairment checklist in people with subjective cognitive decline. Int Psychogeriatr.

[61] Xu L, Li T, Xiong L, Wang X, Ismail Z, Fukuda M (2021). Reliability and Validity of the Chinese Version of Mild Behavioral Impairment Checklist in Mild Cognitive Impairment and Mild Alzheimer's Disease. J Alzheimers Dis.

[62] Saari T, Smith EE, Ismail Z (2022). Network analysis of impulse dyscontrol in mild cognitive impairment and subjective cognitive decline. Int Psychogeriatr.

[63] Pépin É, Tanguay N, Roy M-P, Macoir J, Bruneau M-A, Ismail Z, et al. Preliminary Validation Study of the French-Quebec Version of the Mild Behavioral Impairment Checklist. Cogn Behav Neurol. 2022:10.1097. 10.1097/WNN.0000000000000321 36201620

[64] Matsuoka T, Ueno D, Ismail Z, Rubinstein E, Uchida H, Mimura M (2021). Neural Correlates of Mild Behavioral Impairment: A Functional Brain Connectivity Study Using Resting-State Functional Magnetic Resonance Imaging. J Alzheimers Dis.

